# Lipopolysaccharide From *E. coli* Increases Glutamate-Induced Disturbances of Calcium Homeostasis, the Functional State of Mitochondria, and the Death of Cultured Cortical Neurons

**DOI:** 10.3389/fnmol.2021.811171

**Published:** 2022-01-05

**Authors:** Zanda Bakaeva, Natalia Lizunova, Ivan Tarzhanov, Dmitrii Boyarkin, Svetlana Petrichuk, Vsevolod Pinelis, Andrey Fisenko, Alexander Tuzikov, Rinat Sharipov, Alexander Surin

**Affiliations:** ^1^Laboratory of Neurobiology, “National Medical Research Center of Children’s Health”, Russian Ministry of Health, Moscow, Russia; ^2^Department of General Biology and Physiology, Kalmyk State University named after B.B. Gorodovikov, Elista, Russia; ^3^Department of Biology, M.V. Lomonosov Moscow State University, Moscow, Russia; ^4^Institute of Pharmacy, The Sechenov First Moscow State Medical University, Ministry of Health of the Russian Federation, Moscow, Russia; ^5^M.M. Shemyakin and Yu.A. Ovchinnikov Institute of Bioorganic Chemistry, Russian Academy of Sciences, Moscow, Russia; ^6^Laboratory of Fundamental and Applied Problems of Pain, Institute of General Pathology and Pathophysiology, Moscow, Russia

**Keywords:** lipopolysaccharide *E. coli* (LPS), glutamate excitotoxicity, intracellular free Ca^2+^ concentration ([Ca^2+^]_i_), mitochondrial potential (ΔΨm), oxygen consumption rates (OCR), primary neuronal cultures, cell survival

## Abstract

Lipopolysaccharide (LPS), a fragment of the bacterial cell wall, specifically interacting with protein complexes on the cell surface, can induce the production of pro-inflammatory and apoptotic signaling molecules, leading to the damage and death of brain cells. Similar effects have been noted in stroke and traumatic brain injury, when the leading factor of death is glutamate (Glu) excitotoxicity too. But being an amphiphilic molecule with a significant hydrophobic moiety and a large hydrophilic region, LPS can also non-specifically bind to the plasma membrane, altering its properties. In the present work, we studied the effect of LPS from *Escherichia coli* alone and in combination with the hyperstimulation of Glu-receptors on the functional state of mitochondria and Ca^2+^ homeostasis, oxygen consumption and the cell survival in primary cultures from the rats brain cerebellum and cortex. In both types of cultures, LPS (0.1–10 μg/ml) did not change the intracellular free Ca^2+^ concentration ([Ca^2+^]_i_) in resting neurons but slowed down the median of the decrease in [Ca^2+^]_i_ on 14% and recovery of the mitochondrial potential (ΔΨm) after Glu removal. LPS did not affect the basal oxygen consumption rate (OCR) of cortical neurons; however, it did decrease the acute OCR during Glu and LPS coapplication. Evaluation of the cell culture survival using vital dyes and the MTT assay showed that LPS (10 μg/ml) and Glu (33 μM) reduced jointly and separately the proportion of live cortical neurons, but there was no synergism or additive action. LPS-effects was dependent on the type of culture, that may be related to both the properties of neurons and the different ratio between neurons and glial cells in cultures. The rapid manifestation of these effects may be the consequence of the direct effect of LPS on the rheological properties of the cell membrane.

## Introduction

A blood-brain barrier damaged by trauma, stroke, or disease does not represent a reliable barrier to infection (Dando et al., 2014; Brown and Ginsberg, 2019). The presence of infectious agents leads to the apoptosis of neurons, microglia, and microvascular endothelial cells (Braun et al., 2002; Bermpohl et al., 2005; Parthasarathy and Philipp, 2012). The death of neurons and glial cells can have long-term effects, even after antibiotics eliminate the bacteria that caused the infection. While it is established that neurogenesis can be accomplished even an adult brain, complete restoration of the functions of the affected areas does not occur (Nakaguchi et al., 2011). Moreover, bacterial infection can induce the apoptosis of neuronal stem cells, impairing the formation of new neurons in the brain (Hofer et al., 2011). Paradoxically, the bacterial toxin-induced apoptosis of cells of the immune system may have a partly positive effect, preventing the excessive elimination of brain cells by the body’s own immune system (Djukic et al., 2014; Pardon, 2015). Therefore, in order to develop effective methods for preventing the death of brain cells, it is necessary to understand the mechanisms leading to cell death during bacterial infection.

Endotoxins, lipopolysaccharides (LPS) of the outer membrane and the flagellar sheath of gram-negative bacteria, are important virulence factors in bacterial infection (Mook-Kanamori et al., 2011; Chu et al., 2020). LPS is commonly used both in the neuroinflammation *in vitro* models and in the LPS-induced neuroinflammation, depression and the LPS-evoked changes in absence epileptic activity *in vivo* (Kovács et al., 2019; Ali et al., 2020). Neuronal/glial cell cocultures are exposed by LPS to induce microglial proliferation. This leads to the cytodestructive and pro-inflammatory effects of IL-1β, a key cytokine secreted by LPS-activated microglia and astrocytes. Together, these processes *in vivo* contribute to the development of neurodegenerative diseases (O’Brien and Austin, 2019; de Almeida et al., 2020). LPS are ligands for Toll-like receptors (TLRs) of the fourth type (TLR4) on the surface of antigen-presenting cells related to innate immune cells (Bsibsi et al., 2002; Uematsu and Akira, 2006; Mook-Kanamori et al., 2011). Microglia expresses all currently known TLRs (13 in humans and 10 in mice), while astrocytes only express TLR1,2,3 and TLR9. Neurons also express a limited repertoire of these receptors, namely TLR3 and TLR7,8,9; however, there are references to the presence of TLR4 in neurons (Préhaud et al., 2005; Mantani et al., 2011). According to localization in cells, TLRs are divided into those located on the plasma membrane and those located in intracellular compartments. For example, TLR9 is found in endosome membranes (Stevens et al., 2008). The cellular repertoire of TLRs depends on the animal species (Barichello et al., 2013). In the early neonatal period, the ratio between TLRs in the brain can vary significantly depending on the age of the animal (Shi et al., 2013). M. Mattson’s group has shown that cerebral ischemia increases the expression of TLR2 and TLR4 in cultured hippocampal neurons (Tang et al., 2007).

Lipopolysaccharide is able to increase intracellular free Ca^2+^ concentration ([Ca^2+^]_i_) in cultured dorsal neurons of the dorsal ganglion (Hou and Wang, 2001). In bacterial meningitis, the concentration of Glu in the cerebrospinal fluid increases (Guerra-Romero et al., 1993; Ma et al., 2003). Measurements of the activity of glutamine synthase, an enzyme that converts Glu to glutamine, in pneumococcal meningitis have shown significant activation of the enzyme in the cortex (Tumani et al., 2000). If the Glu level was increased by inhibiting glutamine synthase, neuronal apoptosis increased markedly. On the contrary, the inhibition of ionotropic *N*-methyl-D-aspartate (NMDA)-type glutamate receptors (NMDARs) weakened convulsions caused by infection of young rats with pneumococci (Kolarova et al., 2003), which indicates the possible involvement of different types of glutamate receptors and their differing contributions to neuronal excitability and death.

Despite long-term studies of glutamate excitotoxicity, the role of the major excitatory neurotransmitter of central nervous system in brain pathology in bacterial infection is only beginning to be elucidated (Parthasarathy and Philipp, 2012). Antibiotics are still the principal drugs to treat an injured brain against bacterial infection (Giovane and Lavender, 2018). However, antibiotics often have a broader spectrum of action than that taken into account in antibacterial therapy (Garrido-Mesa et al., 2013). Particularly, tetracycline antibiotic minocycline inhibits a key enzyme of the DNA repair system, poly (adenosine diphosphate ribose) polymerase-1, preventing excessive consumption of nicotinamide adenine dinucleotide in oxidized form (Alano et al., 2006), blocks the mitochondrial Ca^2+^ uniporter (Schwartz et al., 2013), and attenuates cytosolic and mitochondrial rises in [Ca^2+^] induced by the activation of the NMDARs in cultured neurons (Abramov and Duchen, 2008; Córdoba et al., 2010).

Glu is a leading factor of neuronal death in traumatic brain injury, stroke, and some neurodegenerative diseases (Weinberger, 2006). In the case of brain damage caused by oxygen-deficiency conditions, trauma, or strokes, zones of the energy-dependent disturbance of the cell metabolism, primarily neurons, the so-called penumbra, develop around the affected areas (Dirnagl et al., 1999). The reason is the large amount of Glu diffused from dead neurons and released from astrocytes due to the reversal of glutamate transporters (Dirnagl et al., 1999; Allen et al., 2004; Malarkey and Parpura, 2008). Long-term exposure to high doses of Glu causes in neuronal cultures a biphasic increase in [Ca^2+^]_i_ (Tymianski et al., 1993; Adamec et al., 1998; Castilho et al., 1998). The second phase of the rise of [Ca^2+^]_i_, the so-called delayed calcium deregulation (DCD), is always synchronized with a profound drop in the mitochondrial transmembrane potential (Vergun et al., 1999; Khodorov, 2004). The proportion of neurons in which DCD was observed is almost linearly correlated with the proportion of neurons that died in a few hours after Glu exposure (Limbrick et al., 1995). The drugs capable of preventing the development of DCD and the drop of ΔΨm exhibit neuroprotective effects by reducing the death of cortical neurons during Glu excitotoxicity (Bakaeva et al., 2020).

Excessive glutamate receptors stimulation, mainly NMDAR, which have the most Ca^2+^ and Na^+^ permeability compared with other types of ionotropic glutamate receptors, leads to the Ca^2+^ and Na^+^ overload of neurons, resulting in the disruption of signaling, metabolic and energy processes, and eventually neuronal death (Choi, 1988; Ankarcrona et al., 1995; Sattler et al., 2000; Zipfel et al., 2000; Khodorov, 2004; Mattson, 2007; Duchen, 2012). The involved processes include the activation of Ca^2+^-dependent proteases, phospholipases, kinases, phosphatases, and nucleases, as well as changes in the structure and activity of a huge variety of proteins, in cytosol, in nuclei, and in organelles, especially mitochondria (Nicholls, 2009; Hagenston and Baing, 2011; Connolly et al., 2018). The consequences of glutamate-induced excess Ca^2+^ uptake and disturbances in cell homeostasis consist of a decrease in the energy functions of mitochondria and their ability to retain factor initiation of the programmed cell death (Duchen, 2012; Smith and Schnellmann, 2012).

We have recently shown that toxic doses of glutamate cause rapid swelling of neurons and change the rheological properties of the plasma membrane (Efremov et al., 2020). Fragments of LPS with a truncated hydrophilic part were able to alter the mechanical properties of the plasma membrane and modulate activity of the plasma membrane proteins (Casado and Ascher, 1998; Korinek et al., 2015), accelerating or slowing toxin-induced cell lysis (Carr and Morrison, 1984). Native (full-length) LPS molecules are able to integrate both into artificial membranes (Nagel et al., 2015) and lymphocyte membranes (Ciesielski et al., 2012). In Klöckner et al. (2014), a decrease in current through hyperpolarization-activated and cyclic nucleotide-regulated channels (HCN1) was found within 8 s after the addition of LPS (50 μg/ml) to cardiomyocytes. The interaction of LPS with the cell membrane can occur rather quickly. The addition of LPS (50 μg/ml) to cardiomyocytes caused a decrease in current through hyperpolarization-activated, HCN1 in 8 s after endotoxin application. The effect was observed only if LPS had direct access to the ion channels.

It was noted above that, with brain injuries and strokes, the blood-brain barrier becomes permeable to LPS. One cannot rule out that, under these conditions, relatively safe Glu concentrations may turn out neurotoxic. Therefore, in the present work, the effect of LPS on glutamate excitotoxicity in primary neuronal cultures from the cerebellum and cerebral cortex of rats was studied. We have found that the short-term (15 min) incubation of cells with LPS prior to and during Glu (33 μM) administration slows down the kinetics of [Ca^2+^]_i_ recovery after the removal of Glu, decreases the maximum respiration rate, and increases neuronal death.

## Materials and Methods

### Materials

Cell culture supplies were obtained from Invitrogen (Thermo Fisher Scientific, Waltham, MA, United States). All other reagents were obtained from Invitrogen or Sigma-Aldrich (Merck, St. Louis, MO, United States).

### Primary Neural Cultures Preparation

Granular neuron cultures were prepared from the cerebellum of Wistar rats 6–7 days old. Briefly, the animals were anesthetized and decapitated; the cerebellum was removed and cleaned from the blood vessel lining. A cell suspension (106 cells/ml) was obtained by treating the tissue with trypsin (10 units/ml) and then dissociated by pipetting, and the destroyed cells were washed out by double precipitation in a centrifuge. The suspension (200 μl) was transferred onto coverslips attached to the wells of 35 mm plastic Petri dishes (MatTek, United States). The slides were precoated with polyethyleneimine (1 mg/ml). One hour later, 1.5 ml of a neurobasal medium containing 2% Supplement B-27 and 0.5 mM L-glutamine as well as 25 mM KCl was added. The cells were kept at 37°C in an atmosphere of 5% CO_2_/95% air at 100% humidity. To suppress the growth of glial cells, arabinoside C (AraC, 10 μM) was added to the culture of granular neurons on Days 2–3.

Primary neuroglial cultures from the rat cortex were prepared in generally the same way. The differences were that the cerebral cortex was removed from the animals on Days 1–2 after birth, the tissue was treated with papain (10 units/ml), and the cultures were grown in media containing 5 mM KCl. No AraC was added to the cells. Cultures of cerebellar and cortex granular neurons were used 8–14 days after planting (8–14 days *in vitro*, 8–14 DIV).

Experiments with animals were carried out in accordance with ethical principles and regulatory documents recommended by the European Science Foundation (ESF) and the Declaration on Animal Welfare and in accordance with the Order of the Ministry of Health and Social Development of Russia No. 708n, dated 23.08.2010 (“On the approval of the rules of laboratory practice”).

### Fluorescence Microscopy Measurements

The measurements of intracellular ion concentrations and transmembrane potentials were carried out on the experimental set up involving an Olympus IX-71 inverted microscope equipped with 20× and 40× fluorite objectives, a Sutter Labmda 10-2 illumination system with a 175-W Xenon lamp (Sutter Instruments, United States), and a CoolSNAP HQ2 CCD camera (Photometrics, United States) controlled by MetaFluor software (Universal Imaging Cor, United States). Off-Line Image analysis was performed using MetaFluor Analyst 5.2 and ImageJ.

Changes in the intracellular concentration of free Ca^2+^ ([Ca^2+^]_i_) were measured by loading the cells with high-affinity (Fura-2PE3 or Fluo-5N) or low-affinity (Fura-FF or X-Rhod-5F) Ca^2+^ fluorescent indicators in order to track both small changes in [Ca2+]i (tens to hundreds of nanomoles/l) as well as significant changes in [Ca^2+^]_i_ (units to tens of micromoles/l). The fluorescent Na^+^ indicator benzofuran isophthalate derivatives (SBFI) was used to measure the intracellular concentration of free Na^+^ ([Na^+^]_i_). Cells were loaded with Ca^2+^ and Na^+^ indicators (4–8 μM, 40–60 min, 37°C) in the form of their acetoxymethyl esters capable of penetrating through the plasma membrane into the cytosol, where they are cleaved by proteases to form Ca^2+^- and Na^+^-sensitive forms. Fura and SBFI fluorescence was excited alternately at 340 ± 5 and 380 ± 4 nm and recorded at 525 nm.

Changes in the transmembrane potential of the inner mitochondrial membrane (ΔΨm) or the plasma membrane (ΔΨp) were registered by staining cells with voltage-sensitive probes: Rhodamine 123 (Rh123, 6.6 μM, 15 min, 37°C) or Bis-(1,3-Dibutylbarbituric Acid)Trimethine Oxonol [DiBAC(C4)3], respectively. The latter was always present in the buffer during fluorescence measurements at a concentration of 0.1 μM. Fluo-5N, Rh123, and DiBAC(C4)3 were excited with 485 ± 5 light, and the fluorescence of 525 ± 25 nm was recorded (dichroic mirror 500 nm). Measurements were carried out at 27–29°C in buffered saline containing: 135 mM NaCl, 5 mM KCl, 2 mM CaCl_2_, 1 mM MgCl_2_, 20 mM 4-(2-hydroxyethyl)-1-piperazineethanesulfonic acid (HEPES), and 5 mM D-glucose; pH 7.4. In Ca^2+^-free solutions, CaCl_2_ was substituted by 0.1 mM EGTA and 2 mM MgCl_2_. The solution was changed by a triple washout with a new solution within <25 s. For partial depolarization of mitochondria and verification of the Ca^2+^ uptake in the presence of Glu, mitochondria were depolarized by application of protonophore carbonyl cyanide p-(trifluoromethoxy) phenylhydrazone (FCCP) (Tymianski et al., 1993; Nicholls and Budd, 2000).

Neuron survival was assessed using morphological and biochemical methods. When using the morphological approach, live and dead cells were counted using a fluorescence microscope after staining their soma and nuclei with Syto-13 (1 μM, 15 min, 37°C) and ethidium homodimer (EthD-1) (2 μM, 15 min, 37°C), respectively. Syto-13 penetrates the cell membrane and binds to nuclear RNA and DNA and to RNA in the cytosol. Syto-13 green fluorescence was excited and recorded using the same filters as used for Rh123. Necrotic cells were counted by the fluorescence of nuclei stained with EthD-1, since this probe penetrates into cells only after the loss of the integrity of the plasma membrane. Red fluorescence EthD-1 was excited at 565 ± 10 nm and recorded at wavelengths above 610 nm with a 585 nm dichroic mirror. Fluorescent micrographs were obtained with an EVOS FL Auto microscope (United States) using the software with the same name. The resulting images were processed using the Image J software. Survival was assessed as the ratio of living to dead cells.

A biochemical assay of the cultured cell survival was performed by a reduction of 3-(4,5-dimethylthiazol-2-yl)-2,5-diphenyltetrazolium bromide (MTT) (0.1 mg/ml) to formazan by the intracellular dehydrogenases (Mosmann, 1983; Denizot and Lang, 1986). Kinetics of formazan formation was measured by light absorption at 550 and 620 nm using a ClarioStar plate reader (BMG Labtech, Germany).

The data were acquired using the MetaFluor software; the data were collected as images of the studied regions and as the Microsoft Excel tables of selected areas. The recorded images were processed using the MetaFluor Analyst software (Universal Imaging Cor, United States) or FIJI (ImageJ) (Schindelin et al., 2012).

Individual and statistical data are presented using Graph-Pad PRIZM as mean ± D [The data was processed in Graph-Pad PRIZM 8.0.1 (GraphPad Software, San Diego) and presented according to statistical rules depending on the type of data distribution].

### Measurements of the Cultured Neurons Oxygen Consumption Rate

Neuronal oxygen consumption rates (OCR, pmol/min) were measured using the Seahorse XF24 Extracellular Flux Analyzer (Seahorse Bioscience, North Billerica, MA, United States), at 37°C, in a cell medium, consisting of 130 mM NaCl, 5 mM KCl, 2 mM CaCl_2_, 1 mM MgCl_2_, 20 mM HEPES, 5 mM Glucose, and 5 mM NaHCO3, at pH ∼7.4. The microplate-based respirometry utilizes a 24-well plate format and quantifies the OCR at different times, following the addition of LPS, glutamate, or their vehicles.

Prior to each experiment, neurons in each well plate were washed twice with 500 μl of the medium. Four wells per plate did not contain neurons, serving as “blank” wells, to control for temperature-sensitive fluctuations in O^2^-sensitive fluorophore emission. Following washing, each well was filled with 525–600 μl of the medium, and the plates were placed in a CO_2_-free incubator (37°C) for 45 min before each set of measurements to further purge CO_2_ and to allow temperature and pH equilibration. The plates were then loaded into the XF24 analyzer, and the sensors were additionally calibrated for 15 min before the first measurement. The respirometry cycle consisted of a 3-min medium mix, a 2-min wait, and a 3-min measurement stage.

The substances of interest, prepared in an assay medium (75 μl), were preloaded into reagent delivery chambers (A–D), and injected sequentially at predesignated intervals. HEPES (20 mM) was included in the medium to ensure pH stability over the 2-h time course of measurements. To evaluate maximum oxygen consumption, carbonyl cyanide p-(trifluoromethoxy) phenylhydrazone (FCCP, 2 μM), rotenone (1 μM), and antimycin A (1 μM) were used. Subsequently, the obtained data was calculated *via* the Seahorse XF Cell Mito Stress Test Report Generator, which automatically calculates and reports assay parameters.

Maximal OCR and spare respiratory capacity were determined as described in Gerencser et al. (2009). The non-mitochondrial oxygen consumption rate (NOCR) is the minimum OCR measured after the antimycin A/rotenone injection. The basal respiration is the difference between the OCR before application of the first agent and NOCR. The maximal respiration is the difference between the OCR amplitude observed in the presence of FCCP and the NOCR. The spare respiratory capacity is the difference between the maximal respiration and the basal one. The difference between the maximum effect of the OCR to the substance and the last value before the addition of this substance was considered the acute response. All data were normalized to the basal level of the respiration rate (OCR just before the application of any agent).

### Preparation of BDP_FL Labeled Lipopolysaccharide *Escherichia coli* O26:B6

Lipopolysaccharide was conjugated with a fluorescent bodipy dye (BDP)-label. The structure of the BDP_FL fluorescent label is presented in Supplementary Figure 1. This dye is identical to BODIPY FL^®^ and has a more stable and brighter fluorescence (higher extinction and quantum yield) compared to fluorescein derivatives.

Spectral properties are as follows: Excitation maximum: 503 nm, Emission maximum: 509 nm, ε = 92000 L⋅mol^–1^ cm^–1^, Fluorescence quantum yield: 0.97 (Lumiprobe Products, 2020).

To a stirred solution of *E. coli* O26:B6 LPS (3.52 mg, ∼0.27 μM for molecular weight 13 kDa) in a mixture of water (300 μL), DMSO (300 μL) and 0.4 M phosphate buffer (41 μL, pH 7.4), a solution of BDP FL NHS ester [Lumiprobe RUS Ltd. (Russia and CIS), 0.43 mg, 1.1 μM] in DMSO (43 μL) was added. The mixture was stirred for 19 h at room temperature, then diluted twice with water, and fractionated on a Sephadex LH-20 column (∼80 mL, eluent - MeCN/water 1:2). Fractions containing labeled LPS were concentrated in a vacuum to a small volume, and the residue was freeze-dried from water. The yield of BDP labeled lipopolysaccharide (BDP-LPS) was 2.79 mg (∼80%).

BDP-LPS contains no salts and a free label. The content of the label (by spectrophotometric assessment, an absorbance at 507 nm for a solution in a 2-propanol/water 1:1 mixture) is 0.044 μM/mg LPS. Since the molecular weight of *E. coli* O26:B6 LPS is estimated as 13000 Da (Mangoni et al., 2008), we can assume that ∼60% of LPS molecules carry one label molecule in the core moiety.

### Flow Cytometry

Isolation of rat peripheral blood mononuclear cells (PBMCs) has been described previously (Bakaeva et al., 2015). Briefly, blood was taken intravitally from the jugular vein of rats. A 14% (weight/volume) ficoll solution was prepared to obtain a final gradient density of 1.087 g/ml to separate the rat cells in a one-step density gradient of ficoll-urografin. Cell viability was determined by trypan blue staining. Diluted (PBS) and defibrinated blood was centrifuged at 1500 *g* for 15 min at room temperature. To concentrate PBMC, the suspension was centrifuged at 300 g for 10 min.

BDP-LPS (10 μg/ml) was added to the suspension of the PBMC (∼10^6^ cells/ml) in the incubation medium, and the analysis was performed 1 and 3 h after BDP-LPS application. Cells were washed out from unbound BDP-LPS by 1 × 1 ml centrifugation (300 *g*, 10 min). Measurements were performed with a NovoCyte laser flow cytometer ACEA Biosciences, Inc., United States. The lymphocyte population was determined by counting events in the direction of the forward (FSC) and side (SSC) scatter of the laser beam (488 nm). In each experiment, 15,000 cells were counted corresponding to the selected FSC and SSC parameters (the total number of such events in each experiment was 41–69 thousand).

### Statistical Analysis

The data analysis was performed in Graph-Pad PRIZM 8.0.1 (GraphPad Software, San Diego). Received data are presented according to statistical rules depending on the type of data distribution. Testing for the normality of the data was carried out using D’Agostino and Pearson test. In the case of a non-parametric distribution, the data were presented as Turkey box-plot histogram or Median ± interquartile range. To compare such data if there are no more than two comparison groups statistically significant differences were determined according to the Mann-Whitney test. When the number of groups was more than two the Kruskal-Wallis test with Dunn’s multiple comparisons test was applied. In the case of a parametric distribution, the data were presented as Mean ± SD or SEM (the number of repetitions is indicated). When comparing more than two groups with a normal distribution of data 1-way ANOVA + Sidak’s multiple comparisons test was used. For comparing the difference in dynamics between groups, two-way ANOVA with repeated measures, followed by Dunnett’s multiple comparisons test was applied. Values of *P* < 0.05 were considered to be statistically significant.

## Results

### Changes in the Intracellular Calcium and Mitochondrial Potential

**FIGURE 1:**
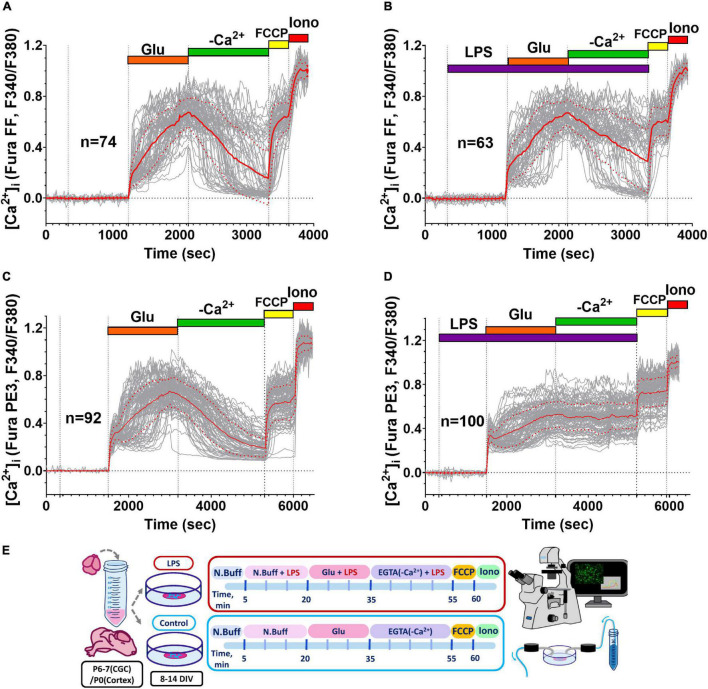
Influence of *E. coli* lipopolysaccharide (LPS) on intracellular free Ca^2+^ concentration ([Ca^2+^]_i_) changes induced by glutamate (Glu) in primary neuronal cultures. **(A,C)** Changes of [Ca^2+^]_i_ induced by Glu (100 μM) alone and **(B,D)** in the presence of (LPS, 10 μM), in cultured neurons of the rat cerebral cortex **(A,B)** and cerebellum **(C,D)**. The results of representative experiments are presented. Glu was co-applied with 10 μM Gly in Mg^2+^-free buffer. [Ca^2+^]_i_ changes are presented as the ratio of fluorescence signals of the low- and high-affinity Ca^2+^ indicators (Fura-FF and Fura-PE3, respectively) excited at 340 and 380 nm (F340/F380); emission was monitored at 525 ± 15 nm. Protonophore FCCP (1 μM) was added in a calcium-free buffer at the end of the experiment to release into the cytosol Ca^2+^ accumulated by mitochondria. The Ca^2+^-selective ionophore ionomycin (Iono, 2 μM, 5 mM Ca^2+^) was employed to calibrate the maximal Ca^2+^ signal of the indicators. The graphs display [Ca^2+^]_i_ responses to Glu of only those neurons in the field of observation, which had delayed calcium deregulation (DCD). Red line corresponds to the mean, and dot red line corresponds to the SD. Schematic of experimental design for panels **A–D (E)**.

Glutamate (Glu, 33 or 100 μM, 10 μM Gly, and 0 Mg^2+^) caused a rapid rise in [Ca^2+^]_i_, which was biphasic in most neurons (Figure 1). Most of the neurons showed a second phase of [Ca^2+^]_i_ increase (delayed calcium deregulation, DCD) in response to a 15-min glutamate exposure (Supplementary Table 1). Cultures of cortical neurons were less resistant to the toxic Glu administration, and cortical cultures had a greater proportion of neurons with DCD, despite a lower Glu concentration (Supplementary Figure 2A). Moreover, DCD developed faster (lag-period of DCD development was shorter) in cultured neurons from a cerebral cortex than in cerebellar neurons (Supplementary Figure 2B). The median of lag-DCD for cortical neurons was 180 s (interquartile range 115–360 s, *n* = 288), whereas, for cerebellar granule cells, the median of lag-DCD was 530 s (interquartile range 290–760 s, *n* = 298). Further, in order to analyze the effect of LPS on the parameters of the two-phase response to excitotoxic doses of glutamate, we focused our study on cortical neurons.

**FIGURE 2:**
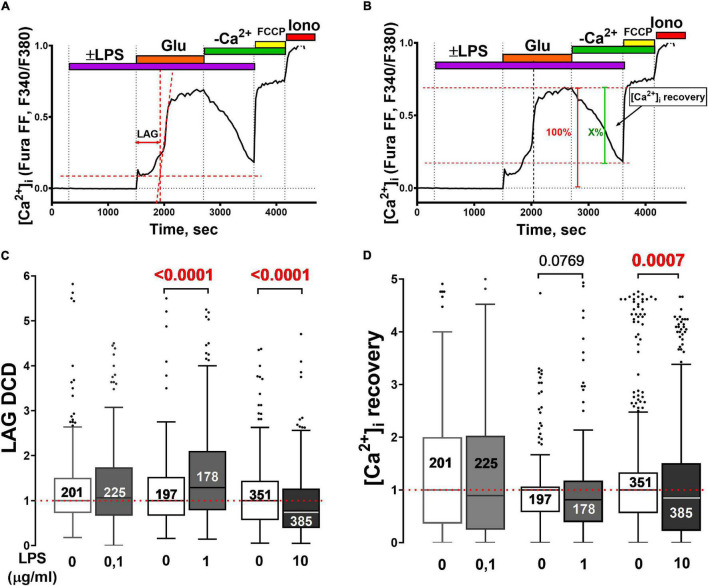
Effect of LPS on the development of glutamate-induced delayed calcium deregulation and post-glutamate [Ca^2+^]_i_ recovery in neuronal cultures from the rat cortex. **(A)** Changes in [Ca^2+^]_i_ in a representative neuron to illustrate the method used to determine the lag-period of delayed calcium deregulation (lag-DCD, s). The onset of DCD is considered to be the intersection of the tangents (red dotted lines) to the [Ca^2+^]_i_ curve during the first phase of the cellular response to Glu (33 μM) and the secondary rise of [Ca^2+^]_i_. The lag-DCD is the time from the Glu addition to the onset of DCD. **(B)** Illustration of a method for quantifying [Ca^2+^]_i_ recovery (X,%) after Glu washout. **(C)** Turkey box-plot histogram of lag-DCD (s) during Glu challenge and **(D)** [Ca^2+^]_i_ recovery (X,%) during Glu washout in the presence of different LPS concentrations. Numbers on histogram bars indicate the amount of neurons that developed DCD; 5–8 experiments for each LPS concentration and control experiments in sister cultures. LPS concentrations (μg/ml) are given beneath the bars. Statistically significant differences were determined according to the Mann-Whitney test.

The LPS (0.1–10 μg/ml) had no effect on [Ca^2+^]_i_ in resting neurons from both the cortex (Figures 1A,B) and cerebellum (Figures 1C,D) and did not significantly increase the proportion of neurons, in which 15-min Glu administration induced the second phase of [Ca^2+^]_i_ increase (delayed Ca^2+^ deregulation, DCD) (Supplementary Data 1). However, LPS influenced the beginning of Glu-induced DCD and slowed down the recovery of [Ca^2+^]_i_ following Glu removal in neurons from the cortex (Figure 2).

Neuronal responses between different cultures may have varied. To take it into account, experiments with LPS were performed in pairs with control sister cultures (glutamate was added without LPS). The lag-DCD values in the control cultures were taken as 1 (arb.un.) to emphasize only those differences that are due to LPS (Figures 2A,C).

Lipopolysaccharide (1 and10 ug/ml) delayed the recovery to low [Ca^2+^]_i_ in the post-glutamate period in cortical neurons Control for LPS 1 ug/ml: median = 1 (interquartile range 0.58–1.06), *n* = 197; LPS 1 ug/ml: median = 0.81 (interquartile range 0.39–1.17), *n* = 178 (Mann Whitney test, *p* = 0.08); Control for LPS 10 ug/ml: median = 1 (interquartile range 0.56–1.33), *n* = 351; LPS 10 ug/ml: median = 0.86 (interquartile range 0.23–1.50), *n* = 385 (Mann Whitney test, *p* = 0.0007) (Figures 2C,D). The method to determine the extent of [Ca^2+^]_i_ recovery during the post-glutamate period and mean values are shown in Figures 2B,D.

**FIGURE 3:**
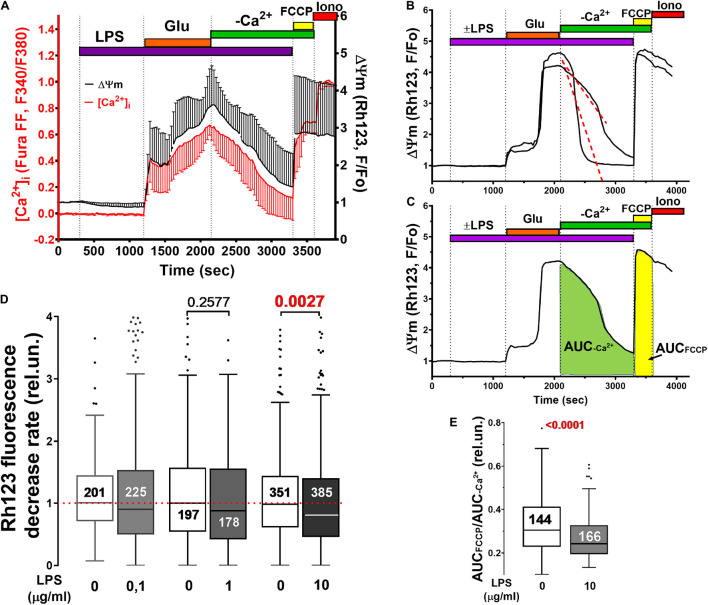
Mitochondrial potential (ΔΨm) changes induced by Glu alone and in the presence of LPS in cultured rat brain cortical neurons. **(A)** Synchronous changes in ΔΨm and [Ca^2+^]_i_ measured with a potential sensitive probe Rh123 and calcium indicator Fura-FF, respectively. [Ca^2+^]_i_ changes are presented as ratio F340/F380 (see legend to Figure 1). Changes in Rh123 fluorescence (excitation: 485 nm; emission: 525 nm) are presented as the ratio F/Fo, where F is the current fluorescence intensity, and Fo is its value at the beginning of the experiment. Curves present Mean ± SD signals of 56 individual neurons. **(B,C)** Illustrate the methods for quantifying ΔΨm recovery during the post-glutamate period. **(D)** The rate of ΔΨm recovery was defined as the slope of the decrease in Rh123 fluorescence following Glu washout (see panel **B**). **(E)** Degree of ΔΨm recovery in each neuron was determined as the ratio of the area under the curve of Rh123 fluorescence (AUC, rel.un.) observed during protonophore FCCP application to the AUC in the post-glutamate period (AUC_*FCCP*_/AUC_–Ca2+_). Numbers on histogram bars indicate the amount of neurons with DCD. Statistically significant differences were determined according to the Mann-Whitney test. Data represent as Turkey box-plot histograms.

Simultaneous measurements of [Ca^2+^]_i_ and mitochondrial transmembrane potential (ΔΨm) showed that LPS did not affect the synchronicity of changes in these parameters during Glu administration (Figure 3A).

A decrease in the Rh123 signal in the post-glutamate period (Figures 3A,B) can be caused by either the restoration of ΔΨm (a decrease in mitochondrial depolarization) and the capture of the probe back to mitochondria, or the leakage of the Rh123 from the cells. LPS reduced the rate of the decrease in Rh123 fluorescence in the post-glutamate period (Figures 3B,D). This phenomenon can be caused by either the restoration of ΔΨm (a decrease in mitochondrial depolarization) and the capture of Rh123 back to mitochondria, or the leakage of the probe from the cells. To check out this assumption, the ratio of the integral fluorescence intensity (Area Under Curve, AUC) of Rh123 after complete mitochondrial depolarization by protonophore FCCP (AUC_FCCP_) to the integral fluorescence intensity of Rh123 in the post-glutamate period (AUC_–Ca2+_) was measured (Figure 3C). The lower the mitochondrial potential, the less Rh123 was retained in the mitochondrial matrix, the lower the increase in Rh123 fluorescence should be in response to the FCCP addition, and the lower the AUC_FCCP_/AUC_–Ca2+_ ratio is. Indeed, LPS decreased the ratio (Figure 3E), which indicates a more rapid Rh123 release from neurons in the post-glutamate period in the presence of LPS.

### Glutamate and Lipopolysaccharide-Induced Changes in Na^+^ Homeostasis and Neuronal Swelling

**FIGURE 4:**
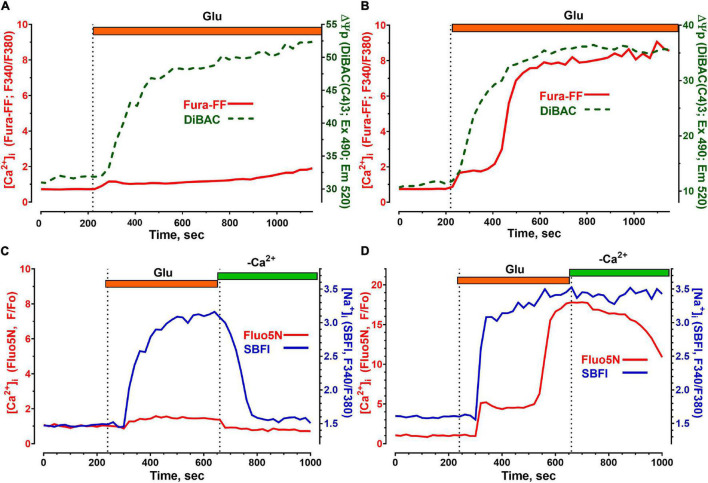
Changes in the concentration of free intracellular Ca^2+^ ([Ca^2+^]_i_), Na^+^ ([Na^+^]_i_) and in the plasmalemmal transmembrane potential (ΔΨp) induced by toxic doses of glutamate in cerebellar neurons. [Ca^2+^]_i_ measurements were performed using low-affinity fluorescent Ca^2+^ indicators Fura-FF **(A,B)** and Fluo-5N **(C,D)** and are given in relative units (F340/F380, Fura-FF) and (F/Fo, Fluo -5N). The fluorescent Na^+^ indicator SBFI was used to measure [Na^+^]_i_ **(C,D)**. Changes in [Na^+^]_i_ are given in relative units (F340/F380). Changes in ΔΨp **(A,B)** were monitored using a DiBAC (C4)3 fluorescent probe; a decrease in ΔΨp causes the accumulation of the probe in the cytoplasm and therefore corresponds to an increase in the DiBAC (C4)3 signal. Voltage-sensitive probe DiBAC (C4)3 was excited with 485 nm and its fluorescence was registered at 525 nm. Glu concentration and buffers were the same as in Figure 1.

Simultaneous measurements of cytosolic ATP concentration ([ATP]), intracellular pH, and [Ca^2+^]_i_ in individual cultured neurons exposed to neurotoxic Glu concentration revealed that the plasma membrane Na^+^/K^+^-ATPase is the major ATP consumer capable to drop [ATP] below 0.2 mM for 5–10 min (Sharipov et al., 2018). Considering this, simultaneous measurements of [Ca^2+^]_i_ and [Na^+^]_i_ were performed, as well as simultaneous measurements of [Ca^2+^]_i_ and transmembrane potential of the plasma membrane (ΔΨp) of cultured cerebellar neurons (Figure 4).

Glu caused equally rapid depolarization of the plasma membrane both in neurons that showed no DCD during the Glu exposure (Figure 4A) and in neurons in which DCD had already developed (Figure 4B). The drop in ΔΨp is caused by the entry of [Na^+^]_i_ into the cytoplasm (Kiedrowski, 1999). The rate of Na^+^ uptake and the amplitude of the [Na^+^]_i_ rise differ insignificantly, both in the neuron that kept [Ca^2+^]_i_ low (Figure 4C) and in the one that could not resist the toxic effects of the Glu that developed DCD (Figure 4D).

The main difference in the kinetics of changes in [Na^+^]_i_ was observed in the post-glutamate period. Neurons that had DCD recovered low [Na^+^]_i_ more slowly than neurons that resisted the toxic effects of Glu. Interestingly, in both types of neurons, the recovery of [Ca^2+^]_i_ outpaced that of [Na^+^]_i_ (Figures 4C,D) in accordance with our recent data (Sharipov et al., 2018). The faster decrease in [Ca^2+^]_i_ compared to [Na^+^]_i_ after Glu washout (Figures 4C,D) is probably due to the fact that Ca^2+^-ATPases of the plasmalemma (as well as endoplasmic reticulum) are capable of pumping ions with a 3–10 times lower [ATP] than Na^+^/K^+^-ATPase (McIntosh et al., 1996).

**FIGURE 5:**
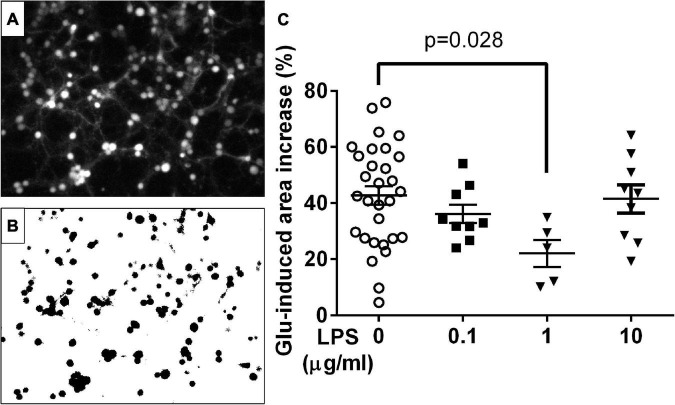
Effect of glutamate (Glu) and LPS on the cell area in the culture of cerebellar granular neurons. **(A)** Typical fluorescent image of cells loaded with Fura-PE3 (excitation: 380 nm; emission: 525 nm). **(B)** Contours around the fluorescent image of the cell obtained using the ImageJ program. **(C)** Changes in the area within cell contours caused by LPS and Glu; the area of each cell was defined as the number of pixels within the contour. Glu and LPS concentrations were 100 μM and 1 μg/ml; the cell culture was exposed to Glu and LPS for 15 and 25 min, respectively. Median ± interquartile range. Statistically significant differences were determined according to the Kruskal-Wallis test with Dunn’s multiple comparisons test.

The [Na^+^]_i_ influx causes neuronal swelling as a result of increased osmotic pressure inside cells (Orlov et al., 2013). Cell swelling is an important aspect of the functional state, resulting in a decrease in the concentration of substances in the cytosol and subsequent changes in signal transduction, transmembrane transport, and intracellular metabolism (MacAulay et al., 2004). Therefore, we examined the influence of LPS, Glu, and their combined effects on the neuronal soma volume. To this end, we measured the surface area of cells by tracking their fluorescence images and assuming that an increase in the area of fluorescent images of cells reflects an increase in their volume. Fluorescence images of Fura-FF loaded neurons were used to monitor changes in [Ca^2+^]i and to track cell swelling, as described recently (Efremov et al., 2020). The results obtained in two of the typical experiments employing the culture of cerebellar granular neurons are shown in Figure 5.

Glutamate increased the total cell area by 42.7 ± 3.3% (Mean ± SEM, *n* = 30 experiments), which corresponds to 1.7-fold increase in volume supposing the soma to be spherical. In the presence of LPS (1 ug/ml) Glu-induced increase of the total cell area was significantly lower (22 ± 5%, *p* = 0.028; *n* = 5 experiments) corresponding to 1.35-fold volume increase (Figure 5). Lower or higher LPS concentrations (0.1 and 10 ug/ml) revealed no appreciable effect on Glu-induced cells area increase (*n* = 9 experiments for each concentration).

### Oxygen Consumption by Neuronal Culture

**FIGURE 6:**
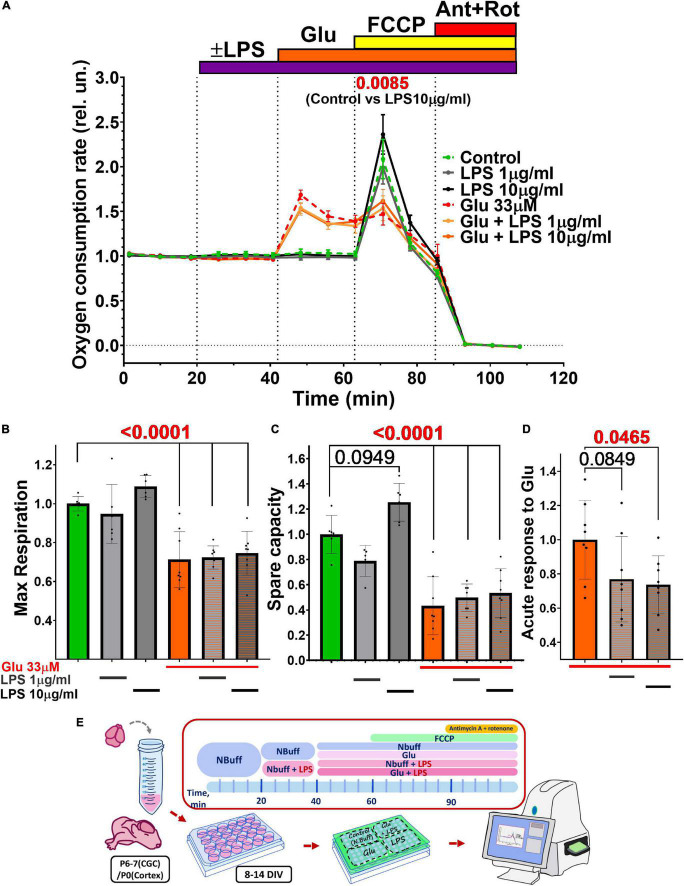
Effect of glutamate (Glu) and lipopolysaccharide (LPS) on the oxygen consumption rate (OCR) by cultured rat cerebral neurons. **(A)** The oxygen consumption rate (OCR) normalized to the culture density in each well of the plate, and then normalized to the basal OCR values (100–200 pmol O2/min) determined during the first 20 min of the experiment run. Agent concentrations were as follows (μM): Glu - 33, protonophore FCCP - 1, inhibitors of the respiratory chain antimycin A (Ant)-0.5, and rotenone (Rot)-0.5. Average values are given for 2 experiments (6 wells). The data are presented as Means ± SEM. Statistically significant differences were found using 2-way ANOVA corrected for multiple comparisons (Dunnett’s multiple comparisons test), LPS 10 μg/ml compared to control. **(B–D)** Effect of LPS and Glu on **(B)** the maximum respiration rate, **(C)** the spare respiratory capacity, and **(D)** the acute response of OCR to glutamate (33 μM, first 5 min of action). The data are presented as Means ± SD (1-way ANOVA + Sidak’s multiple comparisons test). Calculation of maximal OCR, spare respiratory capacity, and acute response to Glu were performed according to Gerencser et al. (2009) (see the section “Materials and Methods”). Schematic of experimental design for panels **A–D (E)**.

The oxygen consumption rate (OCR) is one of the basic parameters of the mitochondrial functional state (Gerencser et al., 2009; Mookerjee et al., 2017; Connolly et al., 2018; Kumagai et al., 2019). To examine the possible effect of LPS on the functional state of mitochondria, the respiration of the neuronal culture from the brain cortex was measured in the presence of LPS, Glu, and over their combined action (Figure 6).

Lipopolysaccharide did not affect the basal OCR, but at a high concentration (10 μg/ml) significantly increased maximal OCR from 2.09 ± 0.54 (Mean ± SD) to 2.37 ± 0.54 (2-way ANOVA + Dunnett’s multiple comparisons test, *p* = 0,0085) (Figure 6A). Some cases suggest that the spare respiratory capacity (SRC), the difference between maximal and basal OCR, during glutamate excitotoxicity better reflects the bioenergetics of mitochondria and the development of OCD than maximal respiration (Yadava and Nicholls, 2007). SRC also tended to increase in the presence of LPS (10 μg/ml) from 1.00 ± 0.15 in the control to 1.26 ± 0.15 (1-way ANOVA + Sidak’s multiple comparisons test, *p* = 0.095) (Figure 6C).

Glutamate (33 μM) during the first 5 min after addition (“acute response”) caused an approximately twofold increase in OCR compared to the basal level from 0.96 ± 0.06 to 1.69 ± 0.15 (*p* < 0.0001) Twenty-minute incubation of the culture with Glu reduced the maximal rate of mitochondrial respiration by ∼30% from 1 ± 0.04 to 0.71 ± 0.14 (*p* < 0.0001) (Figure 6B). Glu also significantly diminished (*p* < 0.0001) spare respiratory capacity from 1.00 ± 0.15 to 0.78 ± 0.12 (Figure 6C).

Lipopolysaccharide, applied in conjunction with Glu, had no additive effect on glutamate-induced changes in maximal and spare capacity (Figures 6B,C), but the acute response of OCR to Glu was significantly (*p* < 0.05) reduced from 1 ± 0.2 for Glu only to 0.73 ± 0.17 for Glu with LPS 10 ug/ml (Figure 6D).

### Morphological and Biochemical Evaluation of Cell Viability

Delayed calcium deregulation development and the cessation of the cells’ ability to recover to low [Ca^2+^]_i_ after Glu removal causes their subsequent death (Nicholls and Budd, 2000; Khodorov, 2004; Nicholls et al., 2007).

**FIGURE 7:**
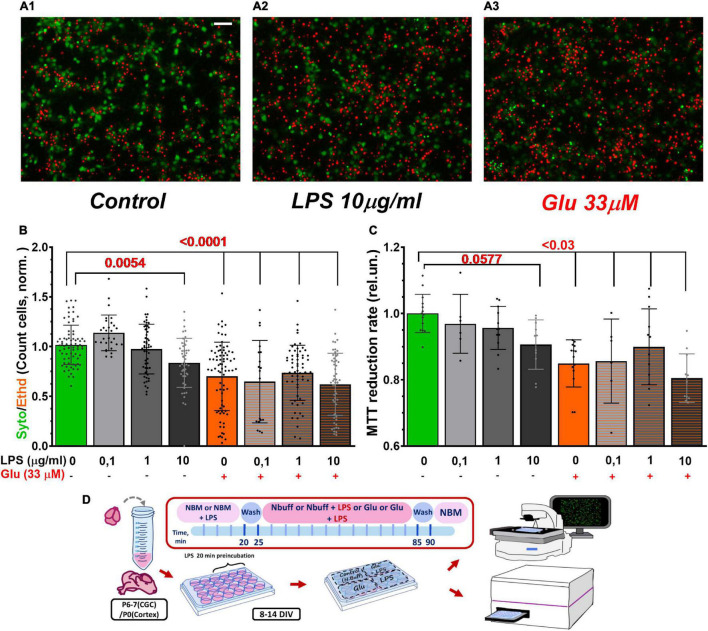
LPS and Glu reduced the survival rate of cultured cerebral neurons evaluated by morphological **(A,B)** and biochemical **(C)** approaches. **(A)** Images of primary neuronal cultures from the rat cortex loaded with the vital fluorescent dyes. Color codes: Syto-13 - green cytoplasm and nucleus of live cells; EthD-1 - red fluorescence of dead cell nucleus. **(A1)** A typical image of a control culture, **(A2)** A culture exposed to LPS, and **(A3)** Culture treated with Glu. LPS and glutamate concentrations are indicated beneath the bars of the histogram. Exposure time of LPS, Glu, and their combined application was 30 min. **(B)** The ratio of the number of cells that had green Syto-13 fluorescence to the number of cells with red EthD-1 fluorescence normalized to the ratio (Syto/EthD) in control cultures. Each bar was obtained by counting 500–700 cells in 5 non-sister cultures (20–60 images for each group). **(C)** MTT assay of cell viability. The MTT to formazan reduction rate is the slope of the kinetic light absorption curve at 550 nm (A550) for the first 10 min since MTT addition; absorbance at 650 nm (A650) was considered background and subtracted from A550. The data are normalized relative to kinetics of light absorbance in the control wells. Scale bar in the A1 image corresponds to 50 μm. 3 non-sister cultures (6–14 wells for each group). The data are presented as Means ± SD (One-way ANOVA + Sidak’s multiple comparisons test). Schematic of experimental design for panels **A–C (D)**.

Counting the number of cells loaded with vital fluorescent dyes (morphological approach) showed that LPS (10 μg/ml) and Glu (33 μm) reduced the survival of cultured cerebral neurons from 1.00 ± 0.20 (mean ± SD) in the control to 0.84 ± 0.25 (*p* = 0.0054) and 0.70 ± 0.34 (*p* < 0.0001) in exposed to LPS and Glu, respectively (Figures 7A,B). The co-application of LPS and Glu did not lead to a noticeable decrease in the proportion of live neurons compared to the action of Glu alone.

To confirm the results of the morphological approach, the biochemical analysis of cell survival, the MTT assay, was also employed (Mosmann, 1983; Denizot and Lang, 1986). Glia, which always contaminates primary neuronal cultures, expresses much fewer ionotropic glutamate receptors than neurons, so glial cells are significantly more resistant to Glu excitotoxicity than neurons (Nedergaard and Verkhratsky, 2012). Image analysis revealed that results of the MTT assay depend on the presence of glial cells, which also reduce MTT, but more slowly than neurons (Surin et al., 2017). The initial rate of formazan formation better reflects the activity of intracellular dehydrogenases in neurons, whereas the final stage of MTT to formazan reduction depends to a greater extent on glia. Therefore, the initial kinetic of tetrazolium reduction to formazan was considered a more adequate index of the intracellular dehydrogenases activity than the conventional measurement of formazan accumulation following a long (usually tens of minutes) incubation of cells with MTT. In cortical cultures, LPS, Glu, and their combination markedly decreased the initial rate of MTT reduction to formazan (Figure 7C).

### Binding of Lipopolysaccharide and Its Fluorescent Derivative to Cultured Cortical Neurons and Lymphocytes

**FIGURE 8:**
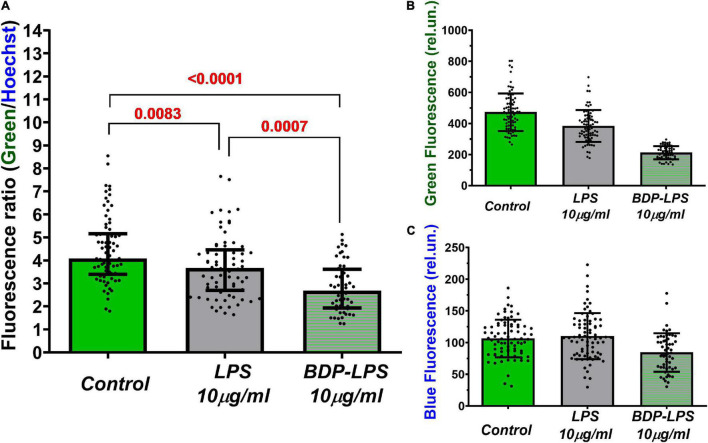
Mean values of green **(A)** and blue **(B)** fluorescence, and the ratio of green/blue fluorescence intensities **(C)** of cultured cortical neurons with or without BDP-LPS incubation (5,5 h). Green autofluorescence was observed in Control and LPS-treated cultures, as well as incubation with a fluorescently labeled analog of LPS (BDP-LPS). The blue fluorescence belongs to the nuclear dye Hoechst33342. BDP-LPS contains a fluorophore BDP in the lipopolysaccharide moiety of LPS (in the O-antigen). Hoechst33342. Green 525 nm fluorescence was excited at 485 nm light. Hoechst33342 blue fluorescence is represented in red for better contrast (excitation: 380 nm; emission: 460 nm). Fluorite lens 20×/NA = 0.70. Images were recorded using Andor NEO CSMOS camera; exposure time: 3200 and 200 ms for green and blue fluorescence, respectively. Median ± interquartile range. Statistically significant differences were determined according to the Kruskal-Wallis test with Dunn’s multiple comparisons test.

In order to determine whether Glu is able to change the distribution of LPS on the cell surface or its endocytosys, a fluorescent analog of LPS containing a BDP-tag in the oligosaccharide moiety (in the O-antigen) (BDP-LPS) was synthesized. LPS (*E. coli* O26: B6) exists in a stock solution (100 μg LPS/ml PBS) as micelles, because its critical micelle concentration is about 14 μg/ml in PBS at pH 7.4 (Santos et al., 2003). Therefore, the fluorescence of the BDP-tag is almost complete self-quenched in the stock solution due to the intention of aromatic fluorescent probes to self-aggregate (Duchen et al., 2003). Accordingly, we expected that the BDP-tag fluorescence would increase upon the incorporation of BDP-LPS into the lipid bilayer due to the dilution with plasma membrane phospholipids, an increase in the intermolecular distance between BDP groups, and their subsequent un-quench. However, exposure of the neuronal culture to BDP-LPS (10 μg/ml) had the opposite effect, namely, a decrease in fluorescence in the region of the spectrum corresponding to the excitation and emission of BDP-LPS (Figure 8 and Supplementary Figure 3). It turned out that BDP-LPS exposure not only did not increase, but reduced green autofluorescence. A similar effect, but to a lesser extent, was observed for cultures incubated with the natural unlabeled LPS (Figure 8).

To minimize the influence of the registration conditions, green fluorescence was normalized to the fluorescence of the same cells stained with the nuclear dye Hoechst33342 (Figure 8 and Supplementary Figure 3). The green/blue fluorescence ratio confirmed the observed effect of the autofluorescence decrease under the exposure to LPS and BDP-LPS (Figure 8C). The nature of the observed phenomenon is not yet clear and, possibly, is due to a change in cellular metabolism induced by the endocytosis of LPS and its fluorescent analog (Malarkey and Parpura, 2008).

### Flow Cytometry

**FIGURE 9:**
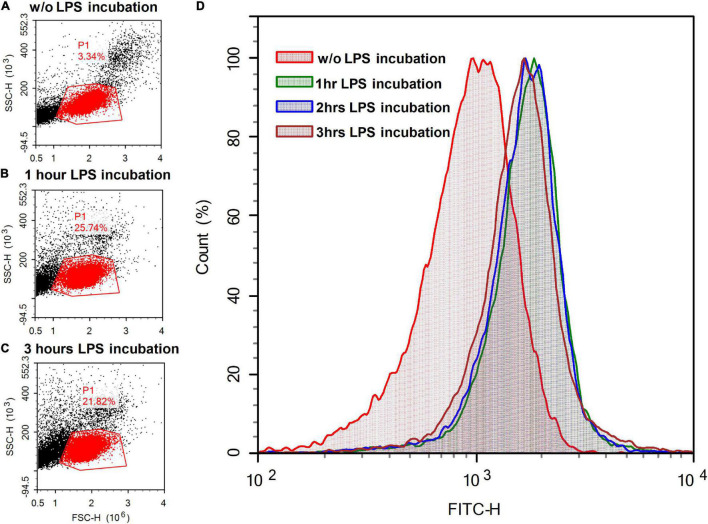
Light scattering (left column of panels) and fluorescent signals (right panel) of lymphocyte suspension **(A)** without (w/o) the addition of fluorescently labeled LPS analog (BDP-LPS) and after **(B)** one and **(C)** 3 h of incubation with BDP-LPS (10 μg/ml). The scattering of laser light (488 nm) in the direction of the beam propagation (FSC-H) and in the perpendicular direction (SSC-H) was recorded for cell selection. Events that had light scattering parameters typical for lymphocytes (gate P1 area) were accumulated (15,000 events) and graphs of the distribution of fluorescent cells were plotted **(D)** (fluorescence was monitored at 535 ± 15 nm).

It has been shown that LPS (100 μg/ml) binds to surface-immobilized TLR4, CD14, and MD-2 for about 1 min (Shin et al., 2007). Biotinylated LPS (5 μg/ml) bound to human monocytes for no more than 5 min (Salomao et al., 2002). To verify that the fluorescent NBD-label did not interfere with the ability of LPS binding to the plasma membrane, we exposed a suspension of rat PBMCs to a fluorescently labeled LPS analog (BDP-LPS) and performed laser flow cytometry. The fraction of the cell population that corresponds to lymphocytes in terms of light scattering in the direction of the laser beam and in the perpendicular direction is outlined by the P1 contour (Figures 9A–C). Incubation of PBMCs with NBD-LPS (10 μg/ml) for 1 h approximately doubled the intensity of green fluorescence compared with the autofluorescence of cells (FITC-H parameter in Figure 9D). Longer incubation (up to 3 h) did not result in a further fluorescence increase (Figures 9B–D).

The structural characteristics of the flow cytometer did not allow us to distinguish the binding of LPS to the cell surface from its uptake *via* endocytosis. However, this data confirms that the increase in the fluorescent signal is, at least partly, caused by binding of LPS to the plasma membrane. Although the obtained data on lymphocytes does not explain the decrease in fluorescence upon the incubation of neuronal cultures in the presence of LPS and BDP-LPS, flow cytometry demonstrates that the fluorescent label does not interfere with the BDP-LPS interaction with cells.

## Discussion

The ability of LPS to activate the immune response is widely used to study the molecular and cellular mechanisms of inflammation both in animals and in cell culture models (Parthasarathy and Philipp, 2012; Barichello et al., 2013). In the present work, employing primary neuronal cultures, we studied whether the bacterial endotoxin LPS can enhance the neurotoxic effect of glutamate, without induction of an inflammatory response. The hypothesis is based on the following. First, LPS is capable not only of specifically interacting with transport proteins and protein complexes on the cell surface (Kirk and Bazan, 2005; Sriram et al., 2015; Vijay, 2018), but, being an amphiphilic molecule with a significant hydrophobic moiety and a large hydrophilic region, LPS can also non-specifically bind to the plasma membrane, altering its properties (Carr and Morrison, 1984; Casado and Ascher, 1998; Korinek et al., 2015). Second, change in the rheological properties of the cell plasma membrane can lead to the increase in cytosolic osmolarity and swelling of the neuronal soma (Efremov et al., 2020), which is observed, for example, in cultured cortical neurons at glutamate administration due to the entry of the large amount of Ca^2+^ and Na^+^ *via* NMDA channels (Kiedrowski, 1999; Sharipov et al., 2018).

The flow cytometry demonstrated (Figure 9) that the increase in the fluorescent signal is, at least partly, caused by binding of LPS to the plasma membrane, and the fluorescent label does not interfere with the BDP-LPS interaction with cells. A decrease in the endogenous green fluorescence of resting neurons in the culture upon the addition of LPS or its fluorescently labeled analog indicates that endotoxin influences intracellular processes—probably a change in the level of NAD(P)H and flavins. Cellular autofluorescence is due to endogenous flavins found within all cells (Benson et al., 1979), but in neurons, mitochondrial FAD^+^ makes the main contribution to green autofluorescence (Shuttleworth, 2010). The quenching of this autofluorescence is consistent with LPS’s effect on the mitochondrial functional state in neurons exposed to glutamate. The local aerobic energy metabolism in the brain varies with changes in the autofluorescence of endogenous compounds, such as NAD(P)H or flavoproteins, and images of cortical neural activity in the rat (Shibuki et al., 2003). It has been shown that LPS can lead to functional ER failure tentatively *via* a mitochondrion-dependent pathway (Kozlov et al., 2009).

Measurements of oxygen consumption by the primary culture of the cortical neurons showed that Glu increased the basal OCR by 50% (Figure 6), obviously due to mitochondrial Ca^2+^ uptake, acceleration of the Krebs cycle, and the activity of the respiratory chain enzymes compensating for the severe increase in ATP demand (Pivovarova et al., 2004; Rueda et al., 2016). Although Glu accelerated the consumption of O_2_, it suppressed the maximum respiratory rate. Similar results were obtained earlier when cultured cortical neurons were stimulated with glutamate or NMDA (Rueda et al., 2016). The reason for the Glu-induced suppression of the maximum OCR has not been clarified, but is probably associated with a decrease in the performance of the respiratory chain enzymes.

Lipopolysaccharide did not affect the basal OCR, but revealed a tendency to increase the maximum respiration rate and the spare respiratory capacity of mitochondria (Figures 6B,C). Possibly, in resting neurons in which the rate of oxygen consumption in the presence of protonophore FCCP has reached a maximum, the addition of LPS increases the efficiency of respiratory chain enzymes. On the other hand, LPS (10 μg/ml) significantly reduced the OCR increase caused by 5-min exposure to Glu (Figure 6D). Apparently, if the activation of the respiratory chain enzymes occurs both due to the ATP consumption and mitochondrial calcium uptake, the effect of LPS on OCR can have the opposite effect. The dependence of OCR on glutamate- and NMDA-induced calcium flux into cortical neurons has been previously shown (Rueda et al., 2016). The change in volume of the neuronal soma should also be taken into account when considering the effects of excitotoxic Glu concentrations in the presence of bacterial endotoxins since the alteration in the properties of the plasma membrane impair the metabolism between mitochondria and the cytosol. The nature of this effect is not yet clear, since the OCR depends on many parameters (Connolly et al., 2018).

Mitochondrial potential measurements showed that, in the presence of LPS, cells retain less of the voltage-dependent fluorescent probe Rh123 (Figure 3). This may be due to a decreased mitochondrial potential and/or an increase in the permeability of the plasma membrane for Rh123. The co-application of LPS (1–10 μg/ml) and Glu did not reveal any additional volume increase of the soma compared with Glu alone. Furthermore, intermediate LPS concentration (1 μg/ml) even diminished Glu-induced increase of the soma volume relative to resting cells from ∼47 to ∼22% (Figure 5C). This indicates that a decrease in ΔΨm is a more probable reason of the reduced Rh123 release from mitochondria caused by FCCP at the end of experiment (Figures 3C,E) than leak of Rh123 during the co-application of LPS and Glu.

Since LPS increased the disturbance of calcium homeostasis, and reduced the increase in the mitochondrial respiration rate induced by Glu, one could expect the effect of LPS on the survival of neurons in culture. Evaluation of the cell cultures’ survival by staining with fluorescent probes Syto-13 and EthD-1 and by the MTT assay showed that, in the cortical cultures, LPS (10 μg/ml) reduced the proportion of live cells, but had no additive effect if co-applied with Glu (33 μM) (Figure 7). LPS and Glu might influence the common link in the neuronal death signal pathway.

The addition of LPS (0.1–1 μg/ml) to primary cultures of the rat cortex astrocytes released free fatty acids, in particular, arachidonic acid (Aizawa et al., 2016). The roles of arachidonic acid in intracellular signaling are extremely diverse and often associated with the modulation of NMDARs (Volterra et al., 1992; Johnson et al., 2019). Exogenous arachidonic acid accelerated the glutamate-induced development of delayed calcium deregulation and profound mitochondrial depolarization in cultured neurons (Surin et al., 2006). Moreover in confirmation, we found that bacterial endotoxin LPS from *E. coli*, while it does not change the basal [Ca^2+^]_i_, but accelerates the beginning of glutamate-induced DCD and slows down the restoration of low [Ca^2+^]_i_ after Glu withdrawal (Figure 2). These effects of LPS depend on the type of culture (different for cultures from cerebellum and cortex) (Figure 1) and may be related to both the properties of neurons and the different ratio between neurons and glial cells in culture (Nedergaard and Verkhratsky, 2012). In neuro-glial cultures from the cortex LPS can enhance the glutamate excitotoxicity influencing the Ca2^+^ homeostasis and the cell death possibly due to increased Ca^2+^ uptake by mitochondria and/or impaired removal of Ca2^+^ and Na^+^ from the cytoplasm. This effects can be masked by a combination of different pathways for Ca2^+^ entry into and out of cells. It was previously shown that LPS can lead to mitochondrial damage, manifested in a violation of the respiratory chain, ROS production, and, as a result, a decrease in ATP synthesis, due to decreased [ATP] in the cytosol. One of the suggested mechanisms is activation *via* the phosphorylation of proteins involved in mitochondrial fragmentation, as well as the influence on the expression of genes encoding proteins involved in mitochondrial fragmentation and ETC components (Lall et al., 2008; Cui et al., 2018; Ma et al., 2018). This is consistent with our data on the effect of LPS on mitochondrial potential (Figures 3D,E).

Thus, it can be summarized that LPS *E. coli* in doses of 10 μg/ml decreases the cell survival of cultured neurons, but with no additive effect if co-applied with Glu. Possibly, LPS and Glu influence the common link in the neuronal death signal pathway. However, we demonstrated that even short-term pre-incubation with LPS affects changes in calcium homeostasis and mitochondrial functional state caused by neurotoxic glutamate concentration. One cannot rule out that these short-term effects of bacterial endotoxin affect intra- and intercellular interactions over a longer period, when LPS triggers long-term mechanisms typical for inflammation.

## Conclusion

**FIGURE 10:**
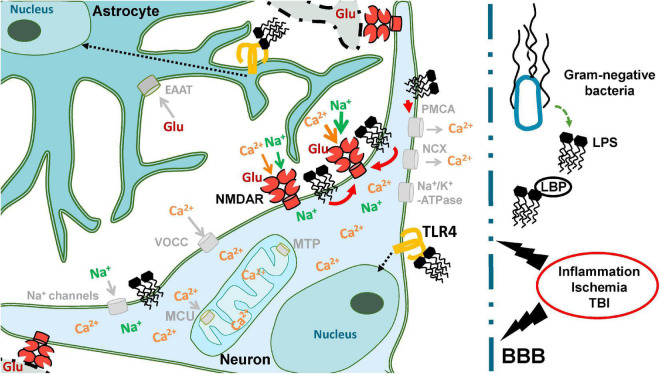
Neuronal ion homeostasis is supported by activity of various protein molecules located in the plasma membrane: ion channels, pumps, secondary transporters. The interaction of LPS with TLR4 can activate intracellular cascades leading to inflammation (black dotted arrows). Concerning the amphiphilic properties LPS is able to influence the rheological properties of plasmalemma and the protein molecules located in it. Our data demonstrates that such effects of LPS could enhance the ionic homeostasis disturbance caused by hyperstimulation of the ionotropic receptors of Glu in case of Ischemia or TBI (red arrows).

We found that bacterial endotoxin LPS from *E. coli* can prevent the restoration of low [Ca^2+^]_i_ after the washout of Glu and enhance the excitotoxic effect of Glu on cultured neurons. The increase in the toxic effect of high doses of Glu is due to the increasing absorption of Ca^2+^ by mitochondria in the presence of LPS and/or a deterioration in the removal of Ca^2+^ and Na^+^ from the cytoplasm. These effects of LPS depend on the type of culture (different for cultures from cerebellum and cortex), which may be related to both the properties of neurons and the ratio between neurons and glial cells in the culture. Considering the received data, the deterioration in the recovery to the initial values of [Ca^2+^]_i_ in the post-glutamate period in neurons exposed to LPS may be associated with the less efficient work of Na^+^/K^+^ -ATPase of the plasma membrane. The reason may lie in a deeper decrease in [ATP] in the cytoplasm and, accordingly, a lack of energy supply and/or disturbances in the spatial structure of the Na^+^/K^+^ -ATP-pump caused by the incorporation of LPS into the plasma membrane. The faster decrease in [Ca^2+^]_i_ compared to [Na^+^]_i_ after Glu washout (Figures 4C,D) is probably due to the fact that Ca^2+^ -ATPases of the plasmalemma (as well as endoplasmic reticulum) as known are capable of functioning at 3–10-fold lower ATP levels than Na^+^/K^+^ -ATPase (McIntosh et al., 1996). The Figure 10 as the scheme illustrating the major findings on LPS action.

## Data Availability Statement

The original contributions presented in the study are included in the article/Supplementary Material, further inquiries can be directed to the corresponding author.

## Ethics Statement

The studies were reviewed approved by the Ethics Committee of the Institute of General Pathology and Pathophysiology (Baltiyskaya st., 8, Moscow, Russia, 125315). Experiments with animals were carried out in accordance with ethical principles and regulatory documents recommended by the European Science Foundation (ESF) and the Declaration on Animal Welfare and in accordance with the Order of the Ministry of Health and Social Development of Russia No. 708n, dated 23.08.2010 (“On the approval of the rules of laboratory practice”).

## Author Contributions

ZB and AS: conceptualization, validation, formal analysis, writing – original draft preparation, and writing – review and editing. ZB, NL, DB, SP, RS, and AT: methodology. ZB, NL, RS, and SP: software. ZB, NL, and AS: investigation. AS and VP: resources and data curation. NL, IT, RS, and DB: visualization. AS: supervision. AS and AF: project administration. AS, AF, and VP: funding acquisition. All authors have read and agreed to the published version of the manuscript.

## Conflict of Interest

The authors declare that the research was conducted in the absence of any commercial or financial relationships that could be construed as a potential conflict of interest.

## Publisher’s Note

All claims expressed in this article are solely those of the authors and do not necessarily represent those of their affiliated organizations, or those of the publisher, the editors and the reviewers. Any product that may be evaluated in this article, or claim that may be made by its manufacturer, is not guaranteed or endorsed by the publisher.

## Abbreviations

Glu: Glutamate
MCU: Mitochondrial calcium uniporter
MTP: Mitochondrial permeability transition pore
PMCA: Plasma membrane Ca^2+^-ATPase
NCX: Na^+^/Ca^2+^-exchanger
VOCC: Voltage-operated calcium channels
EAAT: Excitatory amino acid transporter
NMDAR: ionotropic glutamate receptor NMDA-type
TLR4: Toll-like receptor 4.
