# Supplementary material for: Lipopolysaccharide From E. coli Increases Glutamate-Induced Disturbances of Calcium Homeostasis, the Functional State of Mitochondria, and the Death of Cultured Cortical Neurons
Source: Front Mol Neurosci. 2022 Jan 5;14:811171. doi: 10.3389/fnmol.2021.811171 (PMC8767065; doi:10.3389/fnmol.2021.811171)
Supplement: Supplementary file 1 [file Image_1.pdf]

Supplementary Material

Supplementary Figures

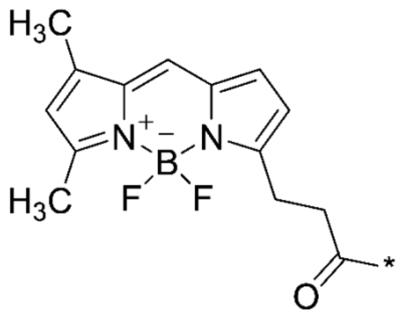

Supplementary Figure 1. The structure of the BDP_FL fluorescent label.

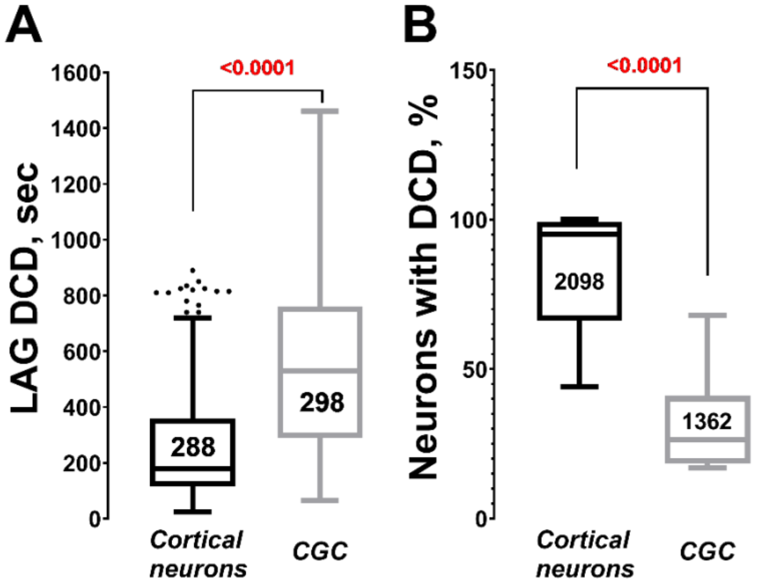

Supplementary figure 2. The time of DCD onset (LAG-DCD, s) (A) and the proportion of neurons (%) with delayed calcium deregulation (DCD) (B) in primary cultures of rat cortical neurons and cerebellar granule cells (CGC). The method used for determining the lag-period of DCD (lag-DCD, s) is shown in **Figure 3A,C**. Numbers on histogram bars indicate the amount of neurons. Statistically significant differences were determined according to the Mann-Whitney test. Data represent as Turkey box-plot histograms.

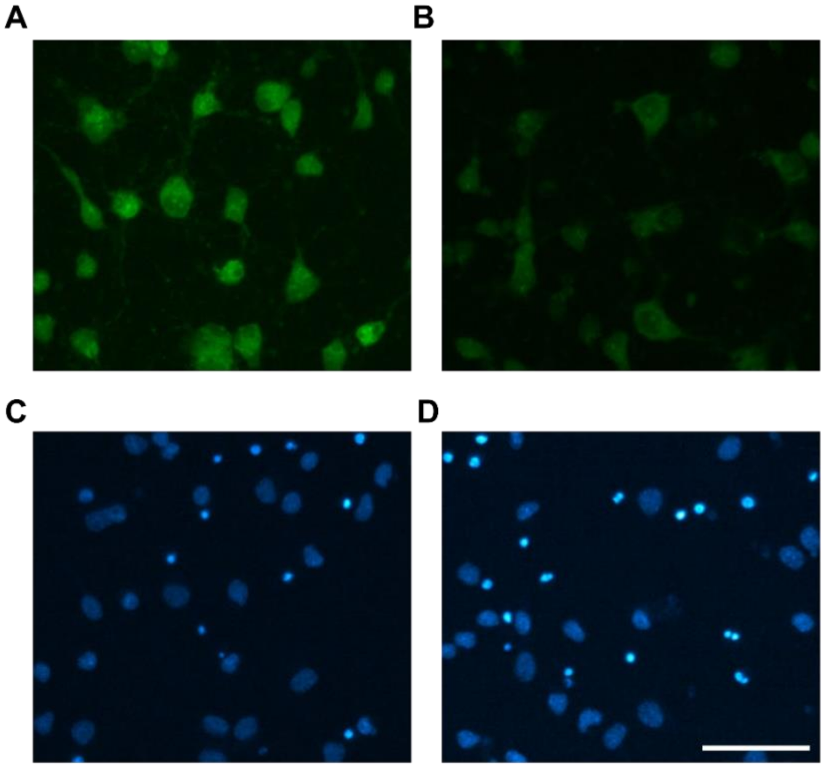

Supplementary figure 3. Fluorescent images of a primary culture of cortical neurons. (A) Green autofluorescence of the cells. (**B**) Green fluorescence of the sister neuronal culture incubated with a fluorescently labeled LPS analogue (BDP-LPS, 10µg/ml, 1 h, 37ºC). (**C**) and (**D**) correspond to the cell cultures in (**A**) and (**B**) after their stain with a nuclear dye Hoechst33342. Green 525 nm fluorescence was excited at 485 nm light. Hoechst33342 is represented in blue (excitation: 380 nm; emission: 460 nm). Fluorite lens 40x/NA = 1.35 oil. Images wererecorded using Andor NEO CSMOS camera; exposure time: 3200 and 200 ms for green and blue fluorescence respectively). Scale bar corresponds to 50 µm.
